# 2-Carboxy­anilinium bromide monohydrate

**DOI:** 10.1107/S1600536809050235

**Published:** 2009-11-28

**Authors:** V. Susindran, S. Athimoolam, S. Asath Bahadur, B. Sridhar

**Affiliations:** a Department of Lighthouses & Lightships, Ministry of Shipping, Nagapattinam Lighthouse & DGPS station, Nagapattinam 611 001, India; b Department of Physics, University College of Engineering Nagercoil, Anna University Tirunelveli, Nagercoil 629 004, India; c Department of Physics, Kalasalingam University, Krishnan Koil 626 190, India; d Laboratory of X-ray Crystallography, Indian Institute of Chemical Technology, Hyderabad 500 007, India

## Abstract

The title compound, C_7_H_8_NO_2_^+^·Br^−^·H_2_O, is isomorphous with 2-carboxy­anilinium chloride monohydrate and contains an intra­molecular N—H⋯O hydrogen bond, forming an *S*(6) motif. The main inter­molecular inter­actions are of the N—H⋯O/Br and O—H⋯O/Br types. Hydrogen-bonding dimers are formed *via* the carboxyl groups and the uncoordinated water mol­ecule, with centrosymmetric *R*_4_^4^(12) ring motifs, in tandem with centrosymmetric *R*_8_^4^(16) ring motifs formed by the cations and bromide anions. The hydrogen-bonded ring motifs inter­sect, forming chains with graph-set motif *C*_4_^3^(10) extending along the *a* axis. These form a two-dimensional hydrogen-bonded network in (101) which is extended along [010] through N—H⋯Br hydrogen bonds. Hydro­philic layers are generated at *z* = 0 and 1/2 which are sandwiched between alternate hydro­phobic layers across *z* = 1/4 and 3/4.

## Related literature

For background to the applications of l-anthranilic acid, see: Anumula (1993 ▶, 1994 ▶); Ma *et al.* (2005 ▶); Prager & Skurray (1968 ▶); Robinson (1966 ▶). For related structures, see: Athimoolam & Natarajan (2006 ▶); Bahadur *et al.* (2007 ▶); Brown & Ehrenberg (1985 ▶); Cinčić & Kaitner 2008 ▶); Zaidi *et al.* (2008 ▶). For hydrogen-bond motifs, see: Bernstein *et al.* (1995 ▶). For a decription of the Cambridge Structural Database, see:Allen (2002 ▶).
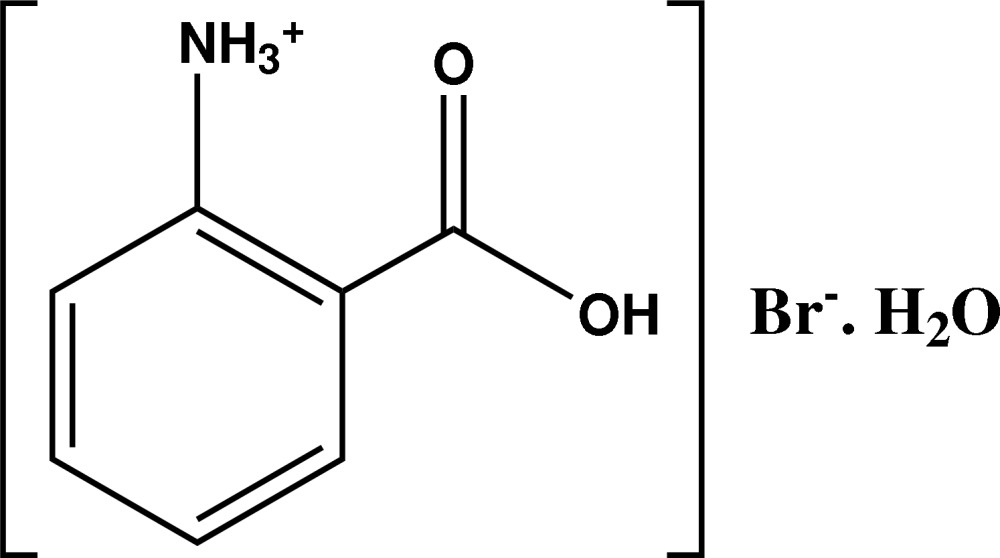


## Experimental

### 

#### Crystal data

C_7_H_8_NO_2_^+^·Br^−^·H_2_O
*M* *_r_* = 236.07
Monoclinic, 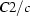

*a* = 23.515 (2) Å
*b* = 4.8923 (4) Å
*c* = 16.5222 (12) Å
β = 91.569 (5)°
*V* = 1900.0 (3) Å^3^
*Z* = 8
Mo *K*α radiation
μ = 4.30 mm^−1^
*T* = 293 K
0.25 × 0.14 × 0.13 mm

#### Data collection

Bruker SMART APEX CCD area-detector diffractometer
Absorption correction: none
7910 measured reflections
1671 independent reflections
1505 reflections with *I* > 2σ(*I*)
*R*_int_ = 0.035

#### Refinement

*R*[*F* ^2^ > 2σ(*F* ^2^)] = 0.031
*wR*(*F* ^2^) = 0.083
*S* = 1.07
1671 reflections
133 parameters
6 restraints
H atoms treated by a mixture of independent and constrained refinement
Δρ_max_ = 0.91 e Å^−3^
Δρ_min_ = −0.44 e Å^−3^

### 

Data collection: *SMART* (Bruker, 2001 ▶); cell refinement: *SAINT* (Bruker, 2001 ▶); data reduction: *SAINT*; program(s) used to solve structure: *SHELXTL/PC* (Sheldrick, 2008 ▶); program(s) used to refine structure: *SHELXTL/PC*; molecular graphics: *ORTEP-3* (Farrugia, 1997 ▶), *Mercury* (Macrae *et al.*, 2006 ▶) and *PLATON* (Spek, 2009 ▶); software used to prepare material for publication: *SHELXTL/PC*.

## Supplementary Material

Crystal structure: contains datablocks global, I. DOI: 10.1107/S1600536809050235/sj2678sup1.cif

Structure factors: contains datablocks I. DOI: 10.1107/S1600536809050235/sj2678Isup2.hkl

Additional supplementary materials:  crystallographic information; 3D view; checkCIF report

## Figures and Tables

**Table 1:** Hydrogen-bond geometry (Å, °)

*D*—H⋯*A*	*D*—H	H⋯*A*	*D*⋯*A*	*D*—H⋯*A*
O2—H2⋯O1*W*	0.85 (3)	1.70 (3)	2.545 (3)	171 (4)
N1—H1*A*⋯Br1^i^	0.89 (2)	2.39 (1)	3.277 (2)	171 (3)
N1—H1*B*⋯Br1^ii^	0.89 (2)	2.44 (2)	3.299 (2)	163 (3)
N1—H1*C*⋯O1	0.89 (2)	1.94 (3)	2.676 (3)	140 (3)
O1*W*—H2*W*⋯O1^iii^	0.83 (3)	2.01 (4)	2.793 (4)	157 (6)
O1*W*—H1*W*⋯Br1	0.82 (3)	2.49 (3)	3.277 (3)	159 (4)

Symmetry codes: (i) 
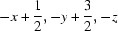
; (ii) 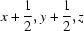; (iii) 
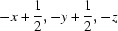
.

**Fig. 1.**
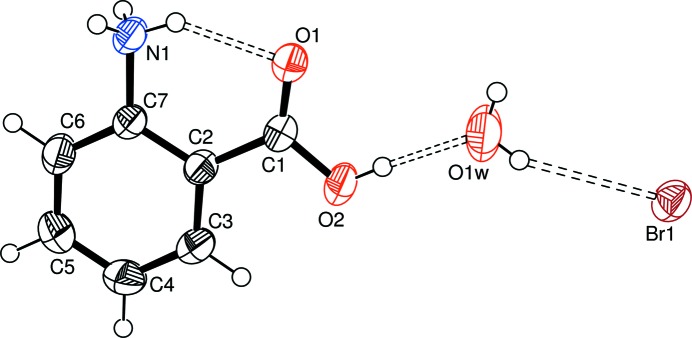
A view of the asymmetric unit of the title compound with the atom labelling scheme. Displacement ellipsoids are drawn at the 50% probability level and H atoms are shown as small spheres of arbitrary radii. Hydrogen bonds are shown as double dashed lines.

**Fig. 2.**
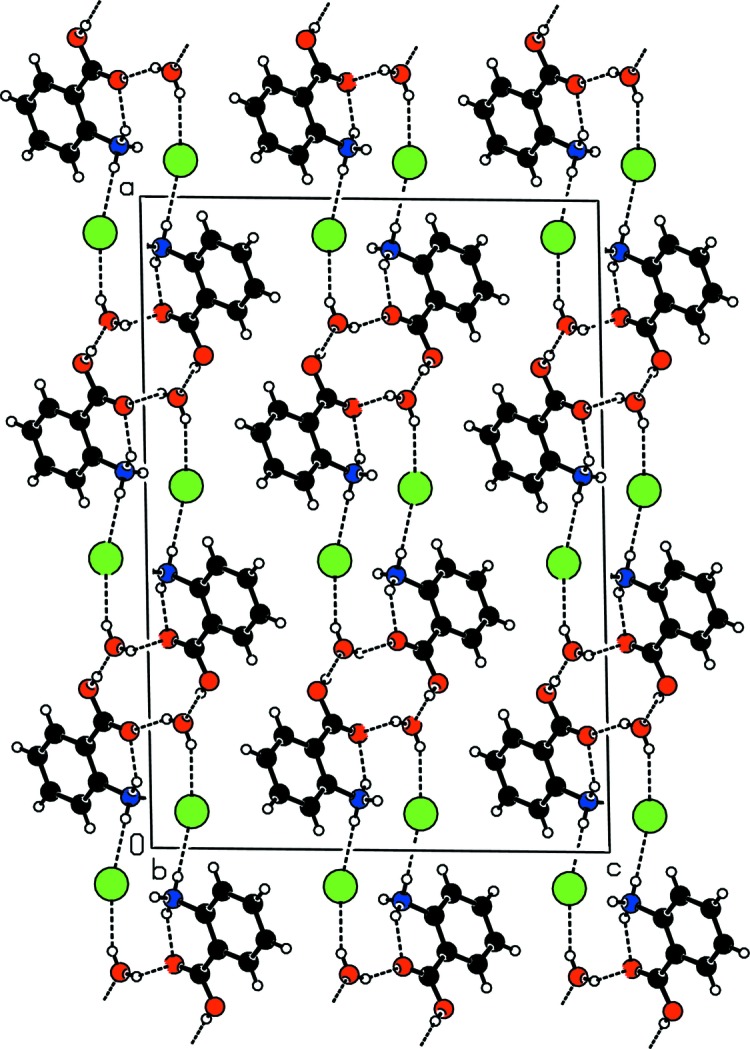
A Packing diagram of (I) viewed along the b axis; Hydrogen bonds are shown as dashed lines. The N1—H1A···Br1(1/2 - x,3/2 - y, -z) H-bond is not shown to avoid overlapping with another N—H···Br bond.

**Fig. 3.**
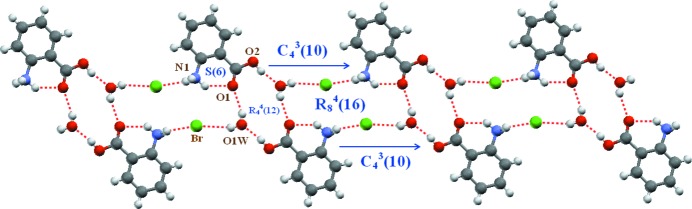
A view of the hydrogen-bonding motifs. Hydrogen bonds are shown as dashed lines.

## supplementary crystallographic information

### Comment

Vitamin *L*, 2-aminobenzoic acid (anthranilic acid), is used as an intermediate for the production of dyes, pigments and saccharin, and its esters are used in preparing perfumes, pharmaceuticals and UV-absorber as well as corrosion inhibitors for metals and mould inhibitors in soya sauce. It is also known to be a specific precursor of the skimmianine and acronidine alkaloids (Prager & Skurray, 1968). Anthranilic acid and its derivatives are used as the preferred fluorescent labels for carbohydrate analysis, with very high sensitivity, and for specific labelling of the reducing mono- and oligosaccharides (Anumula, 1993, 1994). Generally, hydrdoxyl/amino-group-substituted benzoic acid derivatives have active bacteriostatic (*e.g. p*-aminibenzoic acid, a bacterial vitamin) and fragrant properties and are used in the pharmaceutical and perfume industry (Robinson, 1966).

2-Aminobenzoic acid occurs either as a positively or a negatively charged ion or as a neutral molecule (also as a zwitterion), depending on the environment and pH of the solution. The amine group can be protonated, R—NH_3_^+^, (Bahadur *et al.*, 2007) and the carboxyl group can be deprotonated (forming *R*'-CO_2_^-^), where *R* and *R*' are residual moieties. One of the polymorphs of 2-aminobenzoic acid at low temperature occurs as a zwitterion in the solid state (Brown & Ehrenberg, 1985). In our study, anthranilic acid is observed as a protonated carboxyanilinium cation with a bromide anion and hydrogen-bonded water molecule. The present study was undertaken on the isomorphous bromide salt of 2-aminobenzoic acid,(I), to investigate their hydrogen-bonding interactions, aggregation patterns and crystalline packing of the molecules. Recently, an anthranilic acid salt with a chloride anion has been reported (Zaidi *et al.*, 2008). There is only a quantitative change in the crystallographic parameters owing to the size of the anion; the unit cell volume in (I) is about 103 Å^3^ larger than that of the chloride salt (Zaidi *et al.*, 2008). The unhydrated form of 4-aminobenzoic acid - hydrobromic acid crystal was reported by Cinčić & Kaitner, 2008, with the focus on the hydrogen-bonding associations and crystal packing. The structure 2-(methoxycarbonyl)anilinium chloride monohydrate has also been reported (Ma *et al.*, 2005).

The asymmetric unit of the title compound contains a 2-carboxyanilinium cation with a protonated amino group, a bromide anion and a hydrogen-bonded lattice water molecule (Fig. 1). Protonation of the cationic N atom is confirmed by the C—N bond length, 1.464 (3) Å . The asymmetric carboxyl C—O bond lengths (C1—O1 1.216 (3); C1—O2 1.307 (3) indicate the presence of an H atom on O2. The carboxyl group is essentially coplanar with the aromatic ring, with dihedral angle of 2.71 (1)°. However, twisting of the carboxyl plane from the aromatic ring plane is observed in many aminobenzoic acid complexes owing to extensive hydrogen bonding and packing interactions (Athimoolam & Natarajan, 2006).

As aminobenzoic acids have both donor and acceptor sites for hydrogen bonding interactions, they have proved to be versatile reagents for structure extension by linear (chain C motifs) and cyclic (ring *R* motifs) hydrogen-bonding associations, through both the carboxylic acid and amine functional groups (Bernstein *et al.*, 1995). The crystal packing and hydrogen bonding interactions are illustrated in Fig. 2 and hydrogen-bond parameters are listed in Table 2. All ammonium H atoms are involved in hydrogen bonds, two with two different bromide anions and the third with the carbonyl O atom of the same molecule. A strong intramolecular N—H···O hydrogen bond with the graph-set S(6) motif (Fig. 3) is a characterestic feature in many anthranilic acid complexes (Bernstein *et al.*, 1995).

The formation of a classical carboxyl-carboxyl dimer is another of the characteristic features found in most aminobenzoic acid complexes (Cambridge Structural Database, Version 5.29; Allen, 2002), but here the dimerization involves the solvent water molecule. The carboxyl O atom hydrogen bonds with neighbouring water O atom, which further interacts with an inversion-symmetry-related carbonyl O atom (Fig. 3). This generates *R*_4_^4^(12) ring motifs about the inversion centers of the unit cell. Additional centrosymmetrically related hydrogen-bonded rings formed by cation-bromide interactions *via* N—H···Br and O—H···Br hydrogen bonds designated by the graph-set motif *R*_8_^4^(16). These ring motifs are combined and form C_4_^3^(10) chain motifs (Fig. 3) extending along *a* axis of the unit cell. These molecular aggregations form a two-dimensional sheet like structure stacked parallel to the (101) plane of the unit cell (Fig. 2). Further this two-dimensional network is extended to another direction [010] through an N—H···Br (-*x* + 1/2, -*y* + 3/2, -*z*) hydrogen bond. This leads to hydrophilic layers across *z* = 0 and 1/2 which are sandwiched between alternate hydrophobic layers across *z*=1/4 and 3/4, resulting from aromatic ring stacking. Even though the crystalline packing leads to the formation of two weak C—H···O hydrogen bonds [C3—H3···O2^#^, C4—H4···O2^#^; symmetry code: (#) -*x* + 1/2,+*y* + 1/2,-*z* + 1/2], the extensive classical hydrogen bonds predominate. There are no significant C—H···π and π···π interactions.

### Experimental

The title compound was crystallized at room temperature by the slow evaporation technique from aqueous solutions containing 2-aminobenzoic acid (anthranilic acid) with hydrobromic acid in a 1:1 stoichiometric ratio.

### Refinement

All N– and O-bound H atoms are located from difference Fourier map and refined isotropically [N—H = 0.89 - 0.92 (1)Å and O—H = 0.82 (3) - 0.86 (1) Å]. H atoms bonded to C atoms were treated with the riding model approximation, with C—H = 0.93 (aromatic) with *U*_iso_(H) = 1.2*U*_eq_(C).

### Crystal data

**Table:**

C_7_H_8_NO_2_^+^·Br^−^·H_2_O	*F*(000) = 944
*M**_r_* = 236.07	*D*_x_ = 1.650 Mg m^−^^3^
Monoclinic, *C*2/*c*	Mo *K*α radiation, λ = 0.71073 Å
Hall symbol: -C 2yc	Cell parameters from 3371 reflections
*a* = 23.515 (2) Å	θ = 2.8–25.0°
*b* = 4.8923 (4) Å	µ = 4.30 mm^−^^1^
*c* = 16.5222 (12) Å	*T* = 293 K
β = 91.569 (5)°	Needle, colourless
*V* = 1900.0 (3) Å^3^	0.25 × 0.14 × 0.13 mm
*Z* = 8	

### Data collection

**Table:**

Bruker SMART APEX CCD area-detector diffractometer	1505 reflections with *I* > 2σ(*I*)
Radiation source: fine-focus sealed tube	*R*_int_ = 0.035
graphite	θ_max_ = 25.0°, θ_min_ = 1.7°
ω scans	*h* = −27→27
7910 measured reflections	*k* = −5→5
1671 independent reflections	*l* = −19→19

### Refinement

**Table:**

Refinement on *F*^2^	Primary atom site location: structure-invariant direct methods
Least-squares matrix: full	Secondary atom site location: difference Fourier map
*R*[*F*^2^ > 2σ(*F*^2^)] = 0.031	Hydrogen site location: inferred from neighbouring sites
*wR*(*F*^2^) = 0.083	H atoms treated by a mixture of independent and constrained refinement
*S* = 1.07	*w* = 1/[σ^2^(*F*_o_^2^) + (0.0547*P*)^2^ + 0.8797*P*] where *P* = (*F*_o_^2^ + 2*F*_c_^2^)/3
1671 reflections	(Δ/σ)_max_ = 0.001
133 parameters	Δρ_max_ = 0.91 e Å^−^^3^
6 restraints	Δρ_min_ = −0.44 e Å^−^^3^

### Special details

**Table:**

Geometry. All e.s.d.'s (except the e.s.d. in the dihedral angle between two l.s. planes) are estimated using the full covariance matrix. The cell e.s.d.'s are taken into account individually in the estimation of e.s.d.'s in distances, angles and torsion angles; correlations between e.s.d.'s in cell parameters are only used when they are defined by crystal symmetry. An approximate (isotropic) treatment of cell e.s.d.'s is used for estimating e.s.d.'s involving l.s. planes.
Refinement. Refinement of *F*^2^ against ALL reflections. The weighted *R*-factor *wR* and goodness of fit *S* are based on *F*^2^, conventional *R*-factors *R* are based on *F*, with *F* set to zero for negative *F*^2^. The threshold expression of *F*^2^ > σ(*F*^2^) is used only for calculating *R*-factors(gt) *etc*. and is not relevant to the choice of reflections for refinement. *R*-factors based on *F*^2^ are statistically about twice as large as those based on *F*, and *R*- factors based on ALL data will be even larger.

### Fractional atomic coordinates and isotropic or equivalent isotropic displacement parameters (Å^2^)

**Table:**

	*x*	*y*	*z*	*U*_iso_*/*U*_eq_	
C1	0.30710 (11)	0.7182 (6)	0.10059 (17)	0.0402 (6)	
O1	0.32154 (8)	0.5757 (5)	0.04450 (13)	0.0545 (5)	
O2	0.25690 (9)	0.6995 (5)	0.13219 (16)	0.0617 (6)	
C2	0.34468 (10)	0.9244 (5)	0.14078 (15)	0.0363 (5)	
C3	0.32628 (12)	1.0695 (6)	0.20723 (17)	0.0478 (6)	
H3	0.2900	1.0379	0.2261	0.057*	
C4	0.36070 (15)	1.2595 (7)	0.2458 (2)	0.0546 (8)	
H4	0.3474	1.3559	0.2900	0.065*	
C5	0.41512 (14)	1.3072 (6)	0.2189 (2)	0.0533 (8)	
H5	0.4385	1.4345	0.2451	0.064*	
C6	0.43443 (12)	1.1638 (5)	0.15278 (18)	0.0455 (7)	
H6	0.4708	1.1954	0.1342	0.055*	
C7	0.39988 (10)	0.9755 (5)	0.11468 (14)	0.0346 (5)	
N1	0.42208 (10)	0.8318 (5)	0.04455 (15)	0.0392 (5)	
H2	0.2382 (16)	0.565 (7)	0.113 (2)	0.087 (13)*	
H1A	0.4245 (13)	0.955 (5)	0.0050 (15)	0.051 (9)*	
H1B	0.4557 (10)	0.756 (7)	0.055 (2)	0.066 (11)*	
H1C	0.3992 (12)	0.695 (5)	0.0301 (19)	0.048 (9)*	
O1W	0.19167 (12)	0.3305 (6)	0.0733 (2)	0.0938 (11)	
H1W	0.1594 (13)	0.308 (8)	0.090 (3)	0.085 (15)*	
H2W	0.198 (3)	0.214 (9)	0.038 (3)	0.13 (2)*	
Br1	0.056322 (10)	0.18719 (6)	0.089222 (16)	0.04570 (16)	

### Atomic displacement parameters (Å^2^)

**Table:**

	*U*^11^	*U*^22^	*U*^33^	*U*^12^	*U*^13^	*U*^23^
C1	0.0318 (14)	0.0467 (15)	0.0424 (16)	−0.0048 (11)	0.0049 (11)	−0.0007 (12)
O1	0.0413 (10)	0.0646 (12)	0.0581 (12)	−0.0159 (10)	0.0136 (9)	−0.0217 (11)
O2	0.0347 (11)	0.0786 (16)	0.0727 (16)	−0.0194 (10)	0.0183 (10)	−0.0272 (12)
C2	0.0318 (12)	0.0408 (13)	0.0366 (13)	−0.0018 (11)	0.0043 (9)	0.0014 (11)
C3	0.0409 (14)	0.0567 (16)	0.0463 (15)	−0.0042 (13)	0.0129 (12)	−0.0058 (14)
C4	0.061 (2)	0.0586 (17)	0.0442 (17)	−0.0067 (15)	0.0098 (15)	−0.0155 (14)
C5	0.0494 (18)	0.0566 (19)	0.0535 (19)	−0.0104 (13)	−0.0046 (14)	−0.0124 (14)
C6	0.0323 (14)	0.0500 (16)	0.0545 (17)	−0.0055 (11)	0.0040 (12)	−0.0045 (12)
C7	0.0301 (12)	0.0377 (13)	0.0360 (13)	0.0007 (10)	0.0039 (9)	0.0015 (10)
N1	0.0297 (12)	0.0440 (13)	0.0443 (13)	−0.0031 (10)	0.0083 (10)	−0.0025 (10)
O1W	0.0483 (15)	0.101 (2)	0.133 (3)	−0.0328 (15)	0.0277 (17)	−0.062 (2)
Br1	0.0333 (2)	0.0562 (2)	0.0477 (2)	−0.00031 (10)	0.00334 (13)	0.00488 (11)

### Geometric parameters (Å, °)

**Table:**

C1—O1	1.216 (3)	C5—C6	1.386 (4)
C1—O2	1.307 (3)	C5—H5	0.9300
C1—C2	1.486 (4)	C6—C7	1.370 (4)
O2—H2	0.85 (3)	C6—H6	0.9300
C2—C3	1.386 (4)	C7—N1	1.464 (3)
C2—C7	1.401 (3)	N1—H1A	0.890 (18)
C3—C4	1.377 (4)	N1—H1B	0.888 (19)
C3—H3	0.9300	N1—H1C	0.886 (18)
C4—C5	1.386 (5)	O1W—H1W	0.82 (3)
C4—H4	0.9300	O1W—H2W	0.83 (3)
			
O1—C1—O2	122.6 (2)	C4—C5—H5	120.2
O1—C1—C2	123.7 (2)	C7—C6—C5	120.0 (3)
O2—C1—C2	113.7 (2)	C7—C6—H6	120.0
C1—O2—H2	112 (3)	C5—C6—H6	120.0
C3—C2—C7	117.7 (2)	C6—C7—C2	121.4 (2)
C3—C2—C1	120.5 (2)	C6—C7—N1	117.8 (2)
C7—C2—C1	121.9 (2)	C2—C7—N1	120.8 (2)
C4—C3—C2	121.3 (3)	C7—N1—H1A	107 (2)
C4—C3—H3	119.3	C7—N1—H1B	112 (3)
C2—C3—H3	119.3	H1A—N1—H1B	111 (3)
C3—C4—C5	120.1 (3)	C7—N1—H1C	110 (2)
C3—C4—H4	120.0	H1A—N1—H1C	111 (3)
C5—C4—H4	120.0	H1B—N1—H1C	106 (3)
C6—C5—C4	119.5 (3)	H1W—O1W—H2W	109 (5)
C6—C5—H5	120.2		
			
O1—C1—C2—C3	−176.6 (3)	C4—C5—C6—C7	−0.3 (5)
O2—C1—C2—C3	2.2 (4)	C5—C6—C7—C2	0.4 (4)
O1—C1—C2—C7	2.0 (4)	C5—C6—C7—N1	179.3 (3)
O2—C1—C2—C7	−179.3 (3)	C3—C2—C7—C6	−0.6 (4)
C7—C2—C3—C4	0.7 (4)	C1—C2—C7—C6	−179.2 (3)
C1—C2—C3—C4	179.3 (3)	C3—C2—C7—N1	−179.5 (2)
C2—C3—C4—C5	−0.6 (5)	C1—C2—C7—N1	1.9 (4)
C3—C4—C5—C6	0.4 (5)		

### Hydrogen-bond geometry (Å, °)

**Table:**

*D*—H···*A*	*D*—H	H···*A*	*D*···*A*	*D*—H···*A*
O2—H2···O1W	0.85 (3)	1.70 (3)	2.545 (3)	171 (4)
N1—H1A···Br1^i^	0.89 (2)	2.39 (1)	3.277 (2)	171 (3)
N1—H1B···Br1^ii^	0.89 (2)	2.44 (2)	3.299 (2)	163 (3)
N1—H1C···O1	0.89 (2)	1.94 (3)	2.676 (3)	140 (3)
O1W—H2W···O1^iii^	0.83 (3)	2.01 (4)	2.793 (4)	157 (6)
O1W—H1W···Br1	0.82 (3)	2.49 (3)	3.277 (3)	159 (4)

Symmetry codes: (i) −*x*+1/2, −*y*+3/2, −*z*; (ii) *x*+1/2, *y*+1/2, *z*; (iii) −*x*+1/2, −*y*+1/2, −*z*.
